# Pharmacological and mechanistic aspects of quercetin in osteoporosis

**DOI:** 10.3389/fphar.2024.1338951

**Published:** 2024-01-25

**Authors:** Ting-Ting Deng, Wen-Yu Ding, Xi-Xue Lu, Qing-Hao Zhang, Jin-Xin Du, Li-Juan Wang, Mei-Na Yang, Ying Yin, Fan-Jie Liu

**Affiliations:** ^1^ College of Traditional Chinese Medicine, Shandong University of Traditional Chinese Medicine, Jinan, China; ^2^ Shandong Institute of Endocrine and Metabolic Diseases, Jinan, China; ^3^ Endocrine and Metabolic Diseases Hospital of Shandong First Medical University, Shandong First Medical University and Shandong Academy of Medical Sciences, Jinan, China; ^4^ Bone Biomechanics Engineering Laboratory of Shandong Province, Shandong Medicinal Biotechnology Center, School of Biomedical Sciences, Neck-Shoulder and Lumbocrural Pain Hospital of Shandong First Medical University, Shandong First Medical University and Shandong Academy of Medical Sciences, Jinan, China; ^5^ NHC Key Laboratory of Biotechnology Drugs (Shandong Academy of Medical Sciences), Biomedical Sciences College, Shandong First Medical University, Jinan, China; ^6^ Affiliated Hospital of Shandong University of Traditional Chinese Medicine, Jinan, China

**Keywords:** quercetin, antiosteoporosis, pharmacokinetics, toxicology, osteoblast, osteoclast

## Abstract

Osteoporosis (OP) is a bone disease associated with increasing age. Currently, the most common medications used to treat OP are anabolic agents, anti-resorptive agents, and medications with other mechanisms of action. However, many of these medications have unfavorable adverse effects or are not intended for long-term use, potentially exerting a severe negative impact on a patient’s life and career and placing a heavy burden on families and society. There is an urgent need to find new drugs that can replace these and have fewer adverse effects. Quercetin (Que) is a common flavonol in nature. Numerous studies have examined the therapeutic applications of Que. However, a comprehensive review of the anti-osteoporotic effects of Que has not yet been conducted. This review aimed to describe the recent studies on the anti-osteoporotic effects of Que, including its biological, pharmacological, pharmacokinetic, and toxicological properties. The outcomes demonstrated that Que could enhance OP by increasing osteoblast differentiation and activity and reducing osteoclast differentiation and activity via the pathways of Wnt/β-catenin, BMP/SMAD/RUNX2, OPG/RANKL/RANK, ERK/JNK, oxidative stress, apoptosis, and transcription factors. Thus, Que is a promising novel drug for the treatment of OP.

## 1 Introduction

Osteoporosis (OP) is the most prevalent type of systemic bone disease and is characterized by decreased bone mass and damage to the microstructure of the bone tissue, leading to increased bone fragility (Kanis et al., 2019). Patients with OP are more prone to fractures. Over 200 million people worldwide have OP (Palacios, 2022). The lifetime risk of developing the disease in Caucasian women aged >50 years is 50%, and OP-induced fractures can result in substantial medical expenses (up to $25.3 billion by 2025) and impaired physical function (Armas and Recker, 2012).

Nearly all clinical medicine preparations are derived from natural compounds found in plants, animals, insects, marine organisms, and microbes (Harvey et al., 2015). Among them, flavonoids (Rodríguez et al., 2022), alkaloids (Lin et al., 2022), polysaccharides (Lei et al., 2021), quinones (Geng et al., 2019), terpenoids (Bellavia et al., 2021), lignans (Jang et al., 2022), saponins (Siddiqi et al., 2013), polyphenols (Tao et al., 2021), and amino acids (Ling et al., 2021) have been shown to have the ability to inhibit bone resorption and promote bone formation. Quercetin (Que) is a flavonoid compound widely found in traditional Chinese medicines, such as yam bean root, *Scutellaria baicalensis*, and *Sophora japonica*. Its chemical name is 3,3′,4′,5,7 pentahydroxyflavonoid, molecular formula is C15H10O7, and relative molecular mass is 302.236. The appearance is of a yellow acicular crystalline powder, slightly soluble in water, and readily soluble in an alkaline aqueous solution (Yang et al., 2020). Numerous therapeutic effects, such as osteoprotective (Xiao et al., 2023), neuroprotective (Fideles et al., 2023), antiallergic (Mlcek et al., 2016), anti-inflammatory (Hou et al., 2019), anticancer (Rauf et al., 2018), cardiovascular protection (Patel et al., 2018), antiviral (Di Petrillo et al., 2022), antidiabetic (Eid and Haddad, 2017), immunomodulatory (Wang et al., 2021), antihypertensive (Luo et al., 2017), and gastroprotective (Martín et al., 1998), have been reported. Que inhibits bone resorption and promotes bone formation. We reviewed the anti-osteoporosis effects of quercetin and its mechanisms, and summarized its application at the cellular level and in animal experiments in Table 1 and Table 2 respectively.

**TABLE 1:** Use of quercetin in the prevention and treatment of osteoporosis at the cellular level.

Type of cell	Treatment	Findings	References
MSCs	Que (20 μM) + dasatinib (10 nM)	RAD51↑	Wang et al. (2023)
		SASP↓	
MC3T3-E1 induced by iron overload	Que (2.5, 5 μM)	RUNX2, Osterix, ALP, calcium nodules, Bcl-2, SOD, Nrf2, HO-1↑	Xiao et al. (2023)
		Bax, Caspase3, ROS, MDA↓	
BMSCs	Que (5, 25 µM)	RUNX2, ALP, OCN, BMP, β-catenin, Malat1, mineralized nodule↑	Feng et al. (2023)
		NF-κB, p65↓	
hBMSCs	curcumin (1 µM)+ polydatin (10 µM)+quercetin (0.5 µM)	RUNX2, COL1α1, OCN, ALP, miR-98↑	Giordani et al. (2023)
		IL-1β, MCP-1, miR-21, miR-146a, p38-MAPK, p-NF-κB↓	
Senescent BMSCs	Que (20 μM)	calcium nodules↑	Xing et al. (2023)
		SA-β-gal, P16, P21, γH2A, X protein, MCP-1, Cxcl-1, IL-1β, IL-6 ↓	
Raw 264.7 cells	Que (2 μg/mL)	TRAP, CTSK, NFATC-1, ATG5, p62, mTOR, Beclin-1↓	Liu (2022)
hBMSCs	Que (0–10 μmol/L)	cell proliferation, ALP, RUNX2, BGLAP, SPP1, SATB2 ↑	Zhang et al. (2022)
RANKL treated RAW 264.7	Que (200 μM)	cell proliferation, TRAP↓	Zheng et al. (2022)
BMSCs	Que (10 μmol/L)	cell proliferation, ALP, BMP–2, RUNX2, osteocalcin↑	Bian et al. (2022)
		DANCR↓	
MC3T3-E1	Que (10 μmol/L)	cell proliferation, ALP, calcium deposition↑	Ma et al. (2023)
MSCs	Quercetin (0.4–0.6 μg/mL) (in Ti/Gel/CaCO3-Qu-Cr)	ALP, mineralization node formation, P2X7, RUNX2, OSX, OCN, BMP2, Col I, intracellular calcium, ACP↑	Chen et al. (2022)
		ROS↓	
mBMSCs	Que (2, 5 μM)	SOD1, SOD2, p-AMPK, SIRT1, ALP, RUNX2, BMP2, SP7↑	Wang et al. (2021)
		PPARG, FABP4↓	
BMSCs	Que (10 μmol/L)	cell proliferation, ALP, RUNX2↑	Li et al. (2020)
		PPARγ, C/EBPα↓	
	ICI ICI182780 (10 μmol/L) + Que (10 μmol/L)	PPARγ, C/EBPα, RUNX2↑	
		ALP↓	
BMSCs	Que (5, 10 μmol/L)	ALP, BMP-2↑	Bian et al. (2016)
MG-63 cells	Que (0.2 ppm)	cell proliferation, ALP, collagen production, OPG↑	Ruangsuriya et al. (2020)
		RANKL↓	
hBMSCs	quercetin 3-O-β-D-galactopyranoside (25 μM)	cell proliferation, ALP, Extracellular Mineralization calcification, RUNX2, osteorix, OCN, OPN, β-catenin, BMP2, Wnt10a, Wnt10b, Smad1/5, p-ERK↑	Oh et al. (2020)
		perilipin-1, PPARγ, CEBPα, SREBP1c, p-p38, p-JNK, p-c-Fos, p-c-Jun ↓	
BMSCs	Que (1 μmol/L)	calcium nodules, RUNX2, Osterix, β-catenin↑	Yuan et al. (2018)
		NF-κB↓	
hBMSCs	Que (10 μM)	γ-catenin↑ cell proliferation, ALPL, COL1A1, OCN, β-catenin↓	Casado-Díaz et al. (2016)
BMSCs	Que (5 μM)	p-Akt↑	Masuhara et al. (2016)
		TRAP, GPR30↓	
MC3T3-E1	Que (200 μM)	cell proliferation, ALP, RUNX-2, col - 1, ALP, BSP, OC, ARS↑	Tripathi et al. (2015)
osteoblasts	Que aglycone (20 μM)	HO-1, GCLC, CAT, p-ERK1/2, NF-κB p65↑	Messer et al. (2015)
MC3T3-E1	Que (10 μmol/L)	cell proliferation, ALP↑	Mu et al. (2015)
BMSCs	Que (4 μmol/L)	cell proliferation, p-ERK↑	Chen et al. (2014)
Caco-2 cells	Que (100 μM)	VDR, VDR-LBD, TRPV6↑	Inoue et al. (2010)
ROB	Que (0.1, 1, 10 mmol/L)	cell proliferation, mineralized nodule ↑	Yang et al. (2006)
RAW 264.7 cells	Que (0.1–10 μM)	TRAP, OCL number, NF-κB, AP-1↓	Wattel et al. (2004)
peripheral blood monocytic cells		bone resorption, OCL number↑	
MG-63 human osteoblasts	Que (1, 10, 50 μM)	ALP, p-ERK↑	Prouillet et al. (2004)
Rat osteoclasts	Que (2.5, 10, 40 mmol/L)	cell apoptosis ↑ cell proliferation↓	Chen and Wei (2002)

MSCs, mesenchymal stem cells; SASP, senescence-associated secretory phenotype; MC3T3-E1, mouse embryo osteoblast precursor cells; Que, quercetin; RUNX2, Runt-related transcription factor 2; ALP: alkaline phosphatase calcium nodules; Bcl-2, B-cell lymphoma-2; SOD, superoxide dismutase; Nrf2, NF-E2-related factor 2; HO-1:heme oxygenase 1; Bax, Bcl-2, associated X; ROS, reactive oxygen species; MDA, malondialdehyde; BMSCs, bone mesenchymal stem cells; BMP, bone morphogenetic protein; NF-κB, nuclear factor kappa-B; hBMSCs, human bone marrow mesenchymal stem cells; COL1α1, collagen type I alpha 1; miR-98, MicroRNA-98; OCN, osteocalcin; IL-1β, Interleukin-1β; MCP-1, Monocyte Chemoattractant Protein-1; miR-21, MicroRNA-21; miR-146a, MicroRNA-146a; p38-MAPK, p38 Mitogen-Activated Protein Kinase; p-NF-κB, phosphorylated NF-κB; SA-β-gal, a senescence associated beta-galactosidase; P16: multiple tumor suppressor 1; Cxcl-1, CXC-chemokine Ligand-1; TRAP: translocon-associated protein; CTSK, Cathepsin K; NFATC-1, Nuclear Factor Of Activated T-cells, And Cytoplasmic 1; ATG5, Autophagy-related Protein 5; mTOR, mammalian target of rapamycin; BGLAP, osteocalcin; SPP1, secreted phosphoprotein 1; SATB2, Special AT-rich Sequence-binding Protein 2; RANKL, Receptor Activator of NF-KappaB ligand; DANCR, Differentiation Antagonizing Non-protein Coding RNA; P2X7, P2X Ligand-gated Ion Channel 7; Col I, Collagen I; ACP, acid phosphatase; SIRT1, Sirtuin 1; PPARG, peroxisome proliferator-activated receptor gamma; FABP4, Fatty Acid Binding Protein 4; PPARγ, Peroxisome Proliferator-activated Receptor-γ; C/EBPα, CCAAT/enhancer binding proteins alpha; Wnt10a, Wnt Family Member 10a; Wnt10b, Wnt Family Member 10b; p-ERK: Phosphorylated Extracellular Signal-regulated Kinase; SREBP1c, Sterol regulatory element binding protein 1; p-p38, Phosphorylated P38; p-JNK, Phosphorylated C-Jun N-terminal Kinase; p-c-Fos, Phosphorylated c-Fos; p-c-Jun, Phosphorylated C-Jun; hBMSCs, human bone marrow stromal cells; ALPL, alkaline phosphatase, liver; GPR30, G Protein-coupled Receptor 30; ARS, alizarin red-S; GCLC, glutamate cysteine ligase; CAT, catalase; VDR, Vitamin D Nuclear Receptor; VDR-LBD, VDR-ligand-binding Domain; ROB, rat calvarialosteoblasts; TRPV6, transient receptor potential cation channel, subfamily V, member 6; AP-1, Activator Protein-1.

**TABLE 2:** Animal experiments on use of quercetin to prevent osteoporosis.

Animal models	Quercetin dose	Findings	References
OVX-induced osteoporosis mouse model	50 mg/(kg·d)	BV/TV, BMD, Tb.Th, Tb.N↑	Feng et al. (2023)
		Tb.Sp↓	
the iron overload-induced osteoporosis mice model	50, 100 mg/(kg·d)	BV/TV, Tb.Th, Tb.N↑	Xiao et al. (2023)
		SMI↓	
OVX rats	Dasatinib (5 mg/(kg·d)) + Que (50 mg/(kg·d))	femur trabecular bone microarchitecture, BV/TV, trabecular number↑	Wang et al. (2023)
		Tb.Sp, SMI, SnC, p16, p53↓	
radiated (24Gy) C57BL/6 male mice	Dasatinib (5 mg/kg·d) + Que (50 mg/(kg·d))	BV/TV, Conn. Dens, MS/BS,BFR/BS, P1NP↑	Chandra et al. (2022)
		SMI↓	
OVX rats	CaCO3-Qu-Cr (0.4 mg/mL)	BV/TV, Tb.Th, Tb.N, RUNX2, Col I, OCN↑	Chen et al. (2022)
		ROS, TRAP↓	
retinoic acid-induced bone loss model of rats	Que (100 mg/kg·d) +13cRA (80 mg/(kg·d))	Ash content in femur, Ca content in femur, P content in femur, Femoral bone weights, Femur length, BMD, Glutathione content↑	Oršolić et al. (2014)
		MDA↓	
STZ-NA induced diabetic rats	50 mg/(kg·d)	BMD, Tb.Bv/Tb.Tv, Tb.N, Tb.Th, Ct.Th, Tb.Sp↑	Baş and Albeniz (2022)
		SMI↓	
Osteoporosis in Orchiectomy Mice	75, 150 mg/(kg·d)	bone mass, bone strength, bone microstructure, stride length and frequency, insulin-like growth factor-1, high-density lipoprotein, GPRC6A, phospho-AMPK/AMPK↑	Sun et al. (2022)
		phospho-mTOR/mTOR ↓	
OVX rats	50 mg/(kg·d)	urine-Ca, urine-P, BMD, Tb.N, Tb.Th, BV/TV↑	Min et al. (2022)
dexamethasone-induced zebrafish osteoporosis model	12.5, 25 µM	BMA, IOD↑	Hu et al. (2022)
		TNF-α, IL-1β, p65↓	
OVX rats	15 mg/(kg·d)	serum calcium, bone weight, bone volume, trabeculae volume, the total number of osteocytes and osteoblasts, LC3, beclin1, caspase 3 ↑	Vakili et al. (2021)
		total number of osteoclasts, serum osteocalcin, Bcl-2 ↓	
Disuse osteoporosis model in rats	100, 200 mg/(kg·d)	Bone mineral density, trabecular number, trabecular thickness of femur↑ trabecular bone dispersion, ERK1/2, MAPK↓	Wang et al. (2020)
Osteoporosis model in rats with low calcium and high magnesium diet	2.64 μg/(kg·d)	serum-Ca, serum-P, BALP↑	Deng et al. (2020)
		serum-Mg, BGP, CTX1, tPINP↓	
GIO rats	quercetin-loaded transfersomes (10 mg/(kg·d))	femur thickness, density, length, weight, tensile strength of femur bone, serum-Ca, serum-P, ALP↑	Pandit et al. (2020)
		TRAP↓	
OVX rats	50 mg/(kg·d)	Urine-P, urine-Ca, ALP, BMD, Tb.N, Tb.Th, BV/TV↑	Yuan et al. (2018)
OVX rats	100, 200 mg/(kg·d)	BMD, the maximum fracture loading femoral midshaft, fracture loading, stiffness, energy absorption, serum-Ca, serum-P, ALP, P1NP, Osx, RUNX2↑	Xing et al. (2017)
		CTX-1, TRAP, intertrabecular space, p-JNK, p-P38, p-ERK↓	
OVX rats	50, 100, 200 mg/(kg·d)	E2, BMD, U-Ca, U-P, OC, PINP, BALP, BMP2, Smad4↑	Zheng et al. (2017)
		CTX, TRACP-5b↓	
OVX rats	100, 200 mg/(kg·d)	Type I collagen↑ osteocalcin ↓	Feng et al. (2016)
Glucocorticoid-induced osteoporosis (GIO) rats	50, 150 mg/(kg·d)	bone strength, osteocalcin, femoral trabecular, cortical thickness, osteoblast number↑	Derakhshanian et al. (2013)
OVX rats	quercetin-6-C-A-D-glucopyranoside (1, 5 mg/(kg·d))	B.Ar, Cs.Th, T.Ar, T.Pm, B.Pm, B.Pm-T.Ar↑ serum osteocalcin, urinary CTx↓	Siddiqui et al. (2011)
	1, 5 mg/(kg·d)	B.Ar, Cs.Th, T.Ar, T.Pm, B.Pm, B.Pm-T.Ar↑ serum osteocalcin, urinary CTx↓	
OVX rats	75, 150, 300 mg/(kg·d)	ELASTIC, M-STRESS, MLORD, OPG↑	Wang (2009)
		RANKL↓	
OVX rats	75, 150, 300 mg/(kg·d)	BMD↑	Wang (2008)
OVX rats	50, 100, 200 mg/(kg·d)	BMD, BMC↑	Zhu and Wei (2005)
		ACP↓	

OVX, ovariectomized; BV/TV, Bone Volume/Total Volume; BMD, bone mineral density; Tb.Th, Trabecular Thickness; Tb.N, trabecular number; Tb.Sp, Trabecular Separation/Spacing; SMI, skeletal muscle mass index; SnC, senescent cell; p16, multiple tumor suppressor 1; Conn.Dens, connectivity density; MS/BS, mineralizing surface per bone perimeter; BFR/BS, bone formation rate per bone surface; P1NP, Procollagen Type 1 N-terminal Propeptide; RUNX2, Runt-related transcription factor 2; Col I, Collagen I; OCN: osteocalcin; ROS: reactive oxygen species; TRAP, translocon-associated protein; MDA, malondialdehyde; STZ-NA, streptozotocin nicotinamide; Tb.Bv/Tb.Tv, Trabecular volume fraction; Ct.Th, Cortical bone Thickness; GPRC6A, G Protein-coupled Receptor Family C, Group 6, Subtype A; AMPK, AMP-activated Protein Kinase; mTOR:mammalian target of rapamycin; TNF-α, Tumor Necrosis Factor-α; IL-1β, Interleukin-1β; Bcl-2, B-cell lymphoma-2; ERK1/2, Extracellular Signal-Regulated Kinases 1/2; MAPK, Mitogen-Activated Protein Kinase; BALP, bone alkaline phosphatase; BGP, bone gamma-carboxyglutamic-acid-containing proteins; CTX1, type1 collagen; tPINP, N-terminal Peptide; ALP, alkaline phosphatase; TRAP, Tartrate-Resistant Acid Phosphatase; Osx:Osterix; BALP, bone alkaline phosphatase; BMP2, Bone Morphogenetic Protein 2; TRACP-5b, Tartrate-resistant Acid Phosphatase 5b; B.Ar, bone area; Cs.Th, Cortical thickness; T.Ar, trabecular area; T.Pm, Cross-sectional tissue perimeter; B.Pm, Cross-sectional bone perimeter; M-STRESS, maximum stress; M-LORD, mazimum load; BMC, bone mineral content; ACP, acid phosphatase.

## 2 Biological characteristics of Que

### 2.1 Sources of quercetin

Que exists in the flowers, leaves, and fruits of many plants (Wang et al., 2016; Kandemir et al., 2022), mostly in the form of glycosides (Crespy et al., 2001), such as rutin (rutinoside), chrysin, and other plants with high content. Que is a bioactive flavonol found mainly in frequently consumed plant foods such as onions, apples, berries, and broccoli (David et al., 2016). Many herbs, including *Ginkgo biloba*, mulberry leaf, cuscuta, golden buckwheat, forsythia, *Panax ginseng*, and *Fritillaria*, contain Que (Shao et al., 2023). Que exists in the skin and leaves of the Iberian oak *Quercus iberica* of the family Crustacea, the red octopus of the family Berberidaceae, the red drought lily (Hunan forsythia) of the family Hypericum, and the red flax leaves of the family Oleaceae.

### 2.2 Physicochemical properties of Que

**FIGURE 1:**
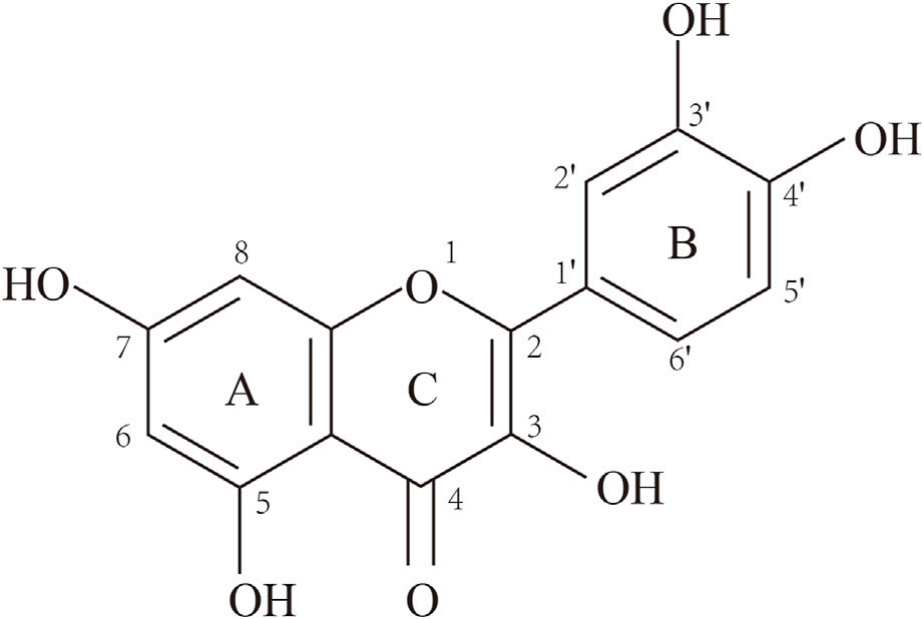
Structure of quercetin (3,3′,4′,5,7-pentahydroxyflavone).

Que is a yellow needle-like crystal (Zou et al., 2021), mostly in the form of glycosides, and can be obtained by acid hydrolysis. Que dihydrate appears as yellow needle-like crystals (dilute ethanol), becomes anhydrous at 95°C–97°C, and has a melting point of 314°C (decomposition). Que is soluble in cold ethanol (1:290), hot ethanol (1:23), methanol, ethyl acetate, glacial acetic acid, pyridine, and acetone. However, it is insoluble in water, benzene, ethyl ether, chloroform, and petroleum ether. The alkaline aqueous solution is yellow and almost insoluble in water, and the ethanol solution tastes very bitter (Wang et al., 2016). The structure of Que is shown in Figure 1.

### 2.3 Biosynthesis of Que

**FIGURE 2:**
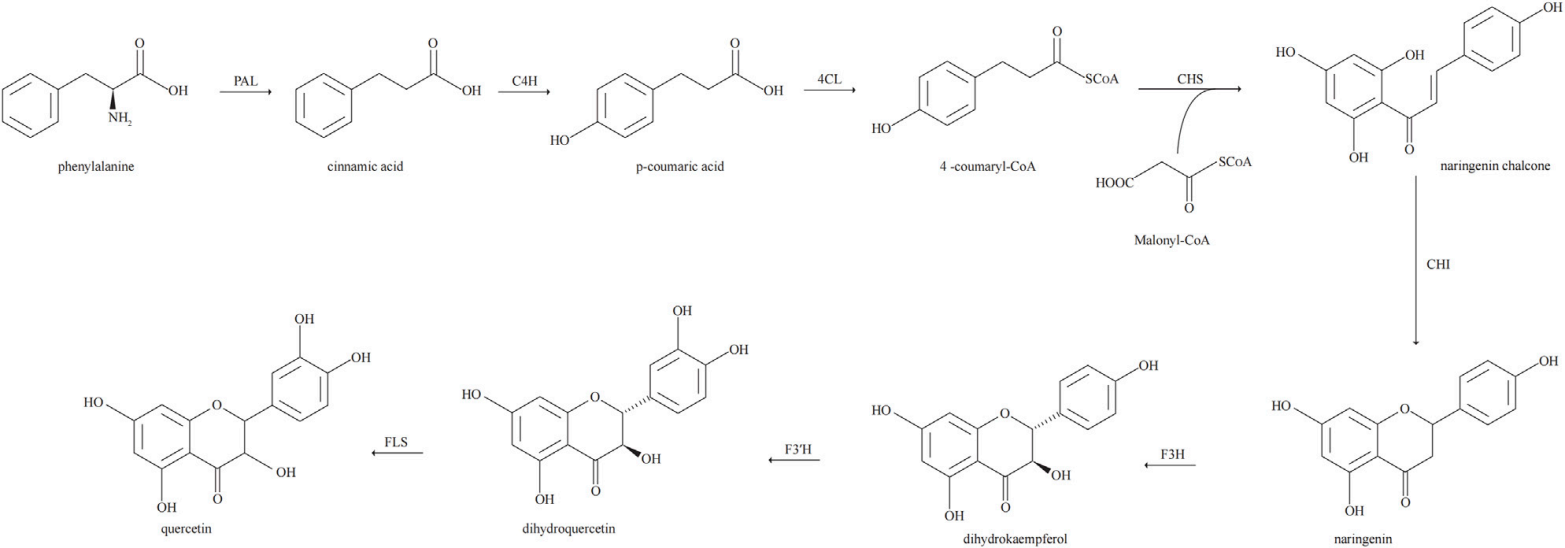
Biosynthetic pathway of quercetin.

Biosynthesis of Que via the phenylpropyl metabolic pathway. In the biosynthesis of Que, phenylalanine derived from the mangiferolic acid pathway is used as the initial precursor to generate cinnamic acid by removing the amino group, catalyzed by phenylalanine ammonia-lyase. Cinnamic acid acquires a hydroxyl group through the catalytic action of cinnamate 4-hydroxylase to form p-coumaric acid. p-Coumaric acid undergoes a thioesterification reaction catalyzed by coumaryl 4-ligase to produce p-coumaroyl coenzyme A. Condensation of one molecule of p-coumaroyl coenzyme A and three molecules of malonyl coenzyme A catalyzed by chalcone synthase produces naringenin chalcone. Naringenin is catalyzed by flavanone 3-hydroxylase to form dihydrokaempferol. Flavanone 3′-hydroxylase is hydroxylated by dihydrokaempferol to form dihydroquercetin. Finally, dihydroquercetin is catalyzed by flavonol synthase for Que biosynthesis (Lakhanpal P and Rai, 2007; Alrawaiq and Abdullah, 2014; Nabavi et al., 2020). The biosynthetic pathway of Que is shown in Figure 2.

## 3 Molecular mechanisms of Que in OP

### 3.1 Mechanism by which quercetin promote osteoblast-mediated bone formation

**FIGURE 3:**
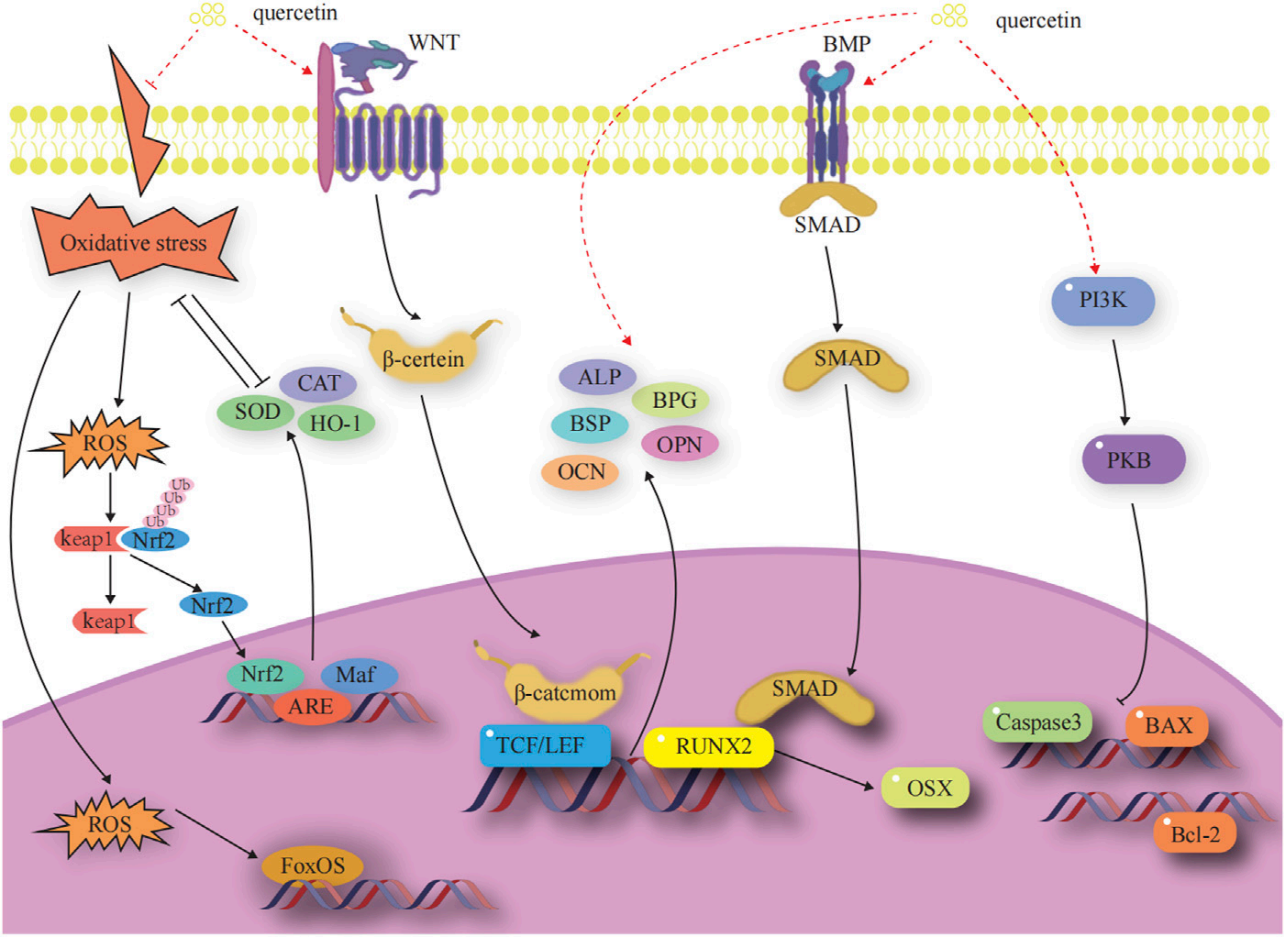
Effects and molecular mechanisms of quercetin induction on osteoblasts. (Little et al., 2002; Nakashima et al., 2002; Steitz et al., 2002; Zhang et al., 2003; Ohri et al., 2005; Kim et al., 2007; Chen et al., 2008; Nusse et al., 2008; Clevers et al., 2014; Jin et al., 2016; An and Shang, 2018; Gao et al., 2018; Zhang et al., 2019; Kim et al., 2021; Kimball et al., 2021; Li et al., 2021; Yu et al., 2021).

Osteoblasts (OBs) undergo four stages of bone formation: proliferation, extracellular matrix maturation, extracellular matrix mineralization, and apoptosis (Marcus and Majumder, 2001). They are affected by a variety of transcription factors and signaling pathways during various stages of development to ultimately complete normal bone formation. The factors and signaling pathways by which Que influences OB-mediated bone formation are outlined below. A diagram of the mechanisms involved in this part of the study are shown in Figure 3.

#### 3.1.1 OB-specific transcription factors

OB-specific Runt-associated transcription factor 2/core-binding factor alpha 1 and special protein 7 transcription factor (osterix) are required for the differentiation of mesenchymal stem cells (MSCs) to OBs and for the formation of functional OBs. The RUNX2 gene is the most critical transcription factor that regulates the differentiation of bone marrow mesenchymal stem cells (BMSCs) into OBs and the maturation of OBs in the process of bone development (Zhang et al., 2003). Expression of the RUNX2 gene is a marker for the onset of OB differentiation, making it the earliest and most specific gene in the process of bone formation. Deletion of this gene will result in hypoplasia or termination of bone development. Osterix acts as a downstream gene of RUNX2 to play an osteogenic role and is expressed exclusively in the cells of the bone tissue, which is required for the differentiation of OBs and for bone formation (Gao et al., 2018). (Figure 3)

Yang (2022) treated mouse bone marrow mesenchymal stem cells (mBMSCs) with Que and found that 5 μM Que significantly upregulates the expression levels of RUNX2 and SP7 mRNA. Xiao et al. (2023) used ferric ammonium citrate (FAC, 200 μM) to construct an iron overload environment. Iron deposition inhibits osteogenic differentiation of MC3T3-E1 cells and suppresses the expression of RUNX2 and Osterix. Iron-overloaded mice have reduced bone mass, loose trabeculae, and thinner bone marrow cavities. Que (2.5 or 5 μM) can rescue the proliferation inhibition of MC3T3-E1 cells induced by FAC and prevent the reduction of RUNX2 and Osterix expression. This action, in turn, rescues the dysfunction of osteogenic differentiation and attenuates the bone loss of MC3T3-E1 cells induced by iron overload.

#### 3.1.2 Wnt/β-catenin signaling pathway

The classical Wnt signaling pathway is an important regulator of bone resorption and formation (Nusse et al., 2008; Clevers et al., 2014). The Wnt signaling pathway is activated when Wnt ligands bind to the heterodimeric receptor molecules of the frizzled protein family and low-density lipoprotein receptor-related protein 5 (LRP5). Subsequently, the Wnt protein binds to the cell surface receptors of the frizzled protein family, which activates the disheveled protein family and the downstream factor GSK-3β. This activation maintains β-catenin in a stable state in the cytoplasm. Eventually, β-catenin enters into the nucleus and interacts with the transcription factor Tcf/Lef to activate the expression of the target genes of Wnt signaling (Little et al., 2002; Nakashima et al., 2002). Dickkopf-1, a secreted glycoprotein, antagonizes Wnt/β-catenin signaling pathway activity by competitively binding to LRP5/6 receptors with Wnt proteins, thereby promoting bone destruction. (Figure 3).

Que promotes osteogenic differentiation of MC3T3-E1 cells by increasing β-catenin protein levels and activating the Wnt/β-catenin pathway (Guo et al., 2017). miR-625-5p expression upregulation or H19 expression downregulation suppresses β-catenin protein levels, and Que promotes the proliferation and osteogenic differentiation of BMSCs by targeting the H19/miR-625-5p axis to activate the downstream Wnt/β-catenin pathway (Bian et al., 2021). However, a high concentration of Que (10 μmol/L) inhibits the Wnt/β-catenin pathway. It downregulates the expression of the cyclin D1 gene involved in the G1/S cell cycle transition in stem cells. Additionally, it decreases the nuclear β-catenin level in undifferentiated MSCs as well as in MSCs induced to differentiate into OBs. The inhibition of the Wnt/β-catenin pathway results in the inhibition of OB formation in MSCs and promotes adipogenesis (Casado-Díaz et al., 2016).

#### 3.1.3 BMP-2/SMADs/RUNX2 signaling pathway

The BMP-2/Smad signaling pathway affects OB differentiation and bone formation (Yu et al., 2021). After the release of BMP-2 in autocrine or paracrine forms, its monomer can form dimers through disulfide bonding. These dimers bind to BMP receptors, regulating the transcription of osteogenic genes through downstream Smad signaling. This process results in the upregulation of RUNX2 and OSX, promoting osteoclast (OC) proliferation and differentiation (Kim et al., 2021). (Figure 3).

Que (2 or 5 μM) increases the relative alkaline phosphatase (ALP) activity and matrix mineralization of mBMSCs and significantly upregulates the mRNA levels of ALP, RUNX2, BMP2, and other OB marker genes, thus promoting the osteogenic differentiation of the third-generation mBMSCs (Yang, 2022). Que enhances the activation of the BMP signaling pathway through the endoplasmic reticulum, upregulates the expression of downstream genes, such as OSX, RUNX2, and OPN, and promotes the proliferation and osteogenic differentiation of BMSCs; crosstalk between BMP-2 and estrogen receptor signaling pathways was experimentally confirmed. The upregulation of RUNX2, OSX, and OPN gene expression by Que and estrogen is inhibited after the addition of ICI182780, an estrogen receptor antagonist (Pang et al., 2018).

#### 3.1.4 Anti-apoptosis-mediated pathways

Apoptosis is a process by which cells respond to physiological and pathological stimulation signals from the environment, changes in environmental conditions, or palliative damage. The Bcl family plays a key role in promoting or inhibiting the intrinsic apoptotic pathway triggered by mitochondrial dysfunction (Zhang et al., 2019). Bcl-2 and Bax are antagonistic proteins involved in the regulation of apoptosis. When Bcl-2 is highly expressed, it promotes the formation of Bax/Bcl-2 heterodimers and inhibits the formation of Bax/Bax homodimers, preventing the release of pro-apoptotic factors, such as cytochrome c. When Bax proteins are highly expressed, they increase the formation of Bax/Bax homodimers and inhibit the formation of Bax/Bax heterodimers. This leads to the activation of the expression of downstream caspase family proteins, promoting the release of apoptotic factors and initiating apoptosis (Chen et al., 2008). (Figure 3).

Que can reduce FAC-induced apoptosis and reactive oxygen species (ROS) production, downregulate the expression of caspase-3 and Bax, and upregulate the expression of Bcl-2 (Xiao et al., 2023; Zhu et al., 2023). Que can attenuate chondrocyte apoptosis by modulating the Bcl-2/Bax-caspase-3 signaling pathway to reduce sodium nitroprusside-induced overproduction of intracellular ROS and restore mitochondrial membrane potential (Hu et al., 2019).

#### 3.1.5 Oxidative stress-mediated pathways

Oxidative stress is a pivotal factor that contributes to the functional uncoupling of OBs and OCs in OP. When a redox imbalance exists in cells, free Nrf2 binds to antioxidant response elements in the nucleus, thereby activating the expression of detoxification genes. Heme oxygenase 1 (HO-1), a well-known phase II detoxification enzyme, inhibits cytotoxicity originating from a variety of oxidative stresses and inflammatory responses, significantly balancing redox homeostasis (Jin et al., 2016). Under oxidative stress, cellular defense mechanisms against oxidative damage are enhanced by upregulating Nrf2 and HO-1 expression (An and Shang, 2018). Members of the forkhead box class O protein (FOXO) family are activated when intracellular reactive oxygen species are increased. FOXO binds β-catenin to shift transcription mediated by the transcription factor T cell factor/lymphocyte enhancer factor in the Wnt pathway toward FOXO transcription to increase the expression of antioxidant enzymes, such as superoxide dismutase (SOD) and catalase (CAT), to attenuate oxidative stress damage, attenuate osteogenic differentiation of BMSCs, and reduce OB bone formation (Kimball et al., 2021). (Figure 3).

Que dose-dependently upregulates HO-1 mRNA expression in BMSCs (Papiez et al., 2008). It significantly enhances Nrf2 nuclear translocation in FAC-induced MC3T3-E1 cells and attenuates FAC-induced oxidative stress injury by activating the Nrf2/HO-1 signaling pathway (Xiao et al., 2023). Que downregulates ROS levels and upregulates the expression of antioxidant genes (Nrf2, CAT, SOD-1, and SOD-2) in BMSCs stimulated by H_2_O_2_ *in vitro*, maintaining the viability of BMSCs and OB differentiation (Lan et al., 2022).

#### 3.1.6 Promotion of bone matrix formation and mineralization

Three non-collagenous proteins, namely, bone salivary protein (BSP), osteopontin (OPN), and osteocalcin (OCN), are essential for the formation and maturation of mineralized tissues. BSP is an effective nucleating agent for hyaluronic acid formation in stabilized agarose gel systems (Steitz et al., 2002). The presence of aspartic acid and phosphoserine in large quantities in the OPN molecule facilitates its binding to the surface of calcium-phosphorus crystals in mineralized tissues and inhibits the calcification and growth of these crystals (Ohri et al., 2005; Li et al., 2021). OCN is involved in the regulation of bone resorption and participates in matrix mineralization processes and OB differentiation. It is associated with bone turnover, maintains the normal rate of bone mineralization, inhibits the rate of cartilage mineralization, and inhibits the formation of abnormal hydroxyapatite crystals in the bone (Kim et al., 2007). (Figure 3).

Que increases BSP transcription in OB-like cells by targeting the reversed CCAAT and FRE elements in the proximal BSP gene promoter (Hunter and Goldberg, 1993). Treatment with Que significantly upregulates the expression of OCN and OPN mRNA in BMSCs, increases the number of mineralized nodules and the accumulation of mineralized matrix in BMSCs, and promotes the osteogenic differentiation of BMSCs (Zhang et al., 2020). Pretreatment with Que significantly restores bone mineralization and OCN mRNA and protein expression levels in lipopolysaccharide (LPS)-inhibited MC3T3-E1 cells in a dose-dependent manner (Guo et al., 2017).

### 3.2 Mechanism by which Que inhibits OC-mediated bone resorption

**FIGURE 4:**
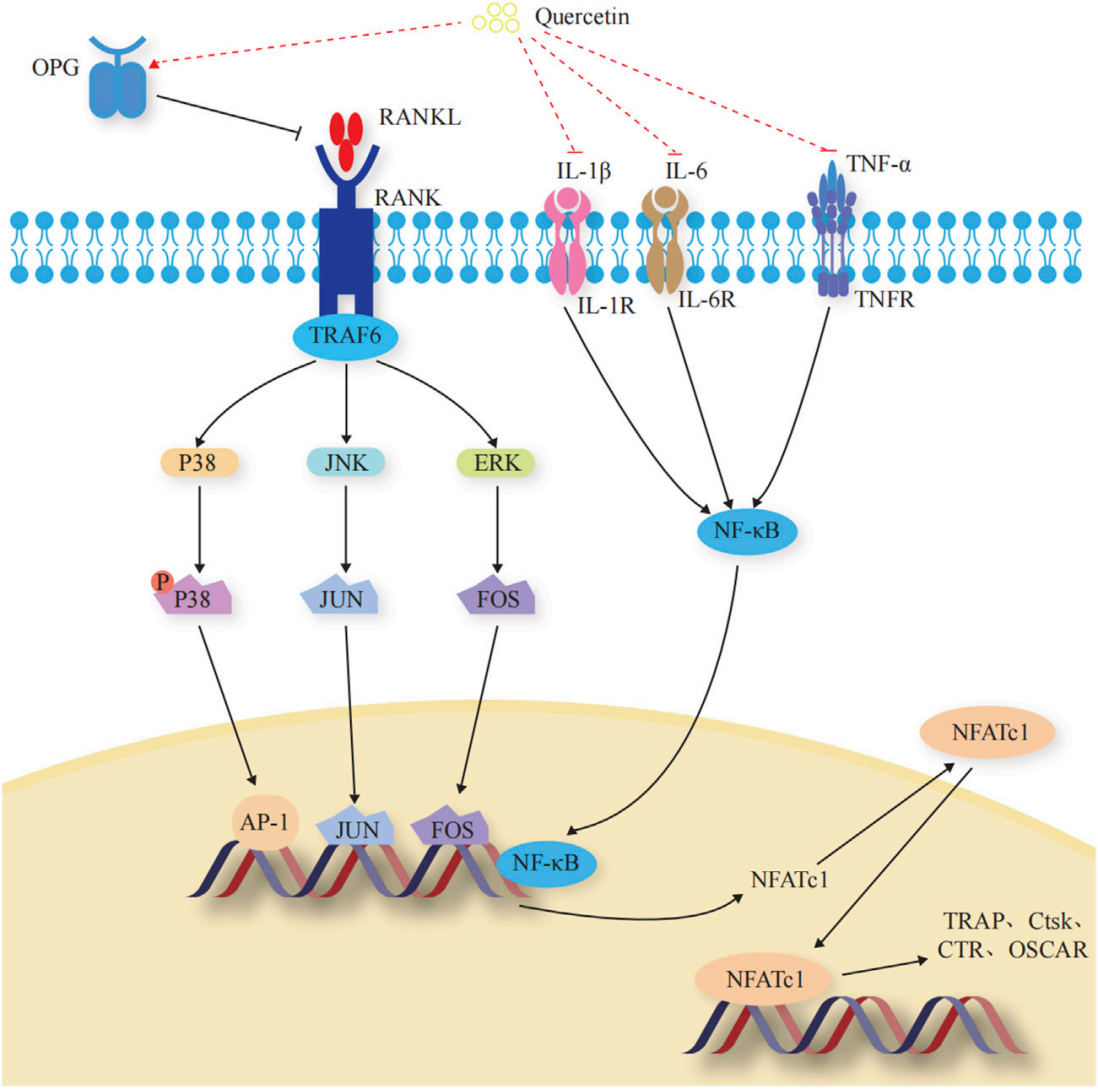
Effects and molecular mechanisms of quercetin induction on osteoclasts. (Takayanagi et al., 2002; Teitelbaum and Ross, 2003; Duplomb et al., 2008; Ikeda et al., 2008; Belibasakis and Bostanci, 2012; Ibrahim et al., 2016; Lee et al., 2016; Pinti et al., 2016; Soysa and Alles, 2016; Amarasekara et al., 2018; Lee et al., 2018; Luo et al., 2018; Noguchi et al., 2018; Ha and Choung, 2020; Kurotaki et al., 2020; Yang et al., 2020; Udagawa et al., 2021; Velletri et al., 2021; Yao et al., 2021).

OCs are multinucleated giant cells with bone resorption functions, formed by the fusion of bone marrow monocyte precursors (Boyce, 2013). They promote bone resorption and remodeling by secreting acids and enzymes that dissolve the bone matrix. When OCs are overactive or in excessive numbers, the equilibrium between OBs and OCs is disrupted, leading to excessive bone resorption, which can also lead to OP. When exercising the function of bone resorption, OCs initially adhere to the surface of the bone matrix to form a closed area, releasing integrins ɑ and β3 to facilitate adherence to the bone matrix. Subsequently, the H+ ion pump with the help of ATP6V0d2 pumps H+ into the closed area, forming an acidic microenvironment to degrade the bone matrix. Meanwhile, OCs secrete tartrate-resistant acid phosphatase (TRAP), matrix metalloproteinase 9 (MMP-9), and cathepsin K (CtsK), among others to dissolve the bone matrix (Soysa and Alles, 2016). The mechanisms underlying the Que regulation of OC-mediated bone resorption are shown in Figure 4.

#### 3.2.1 OC-specific transcription factors

Transcription factors regulate the differentiation and activity of OCs (Kurotaki et al., 2020). Nuclear factor of activated T cell 1 (NFATc1) is the most critical regulator of OC differentiation (Takayanagi et al., 2002). It controls OC-specific genes, such as TRAP, Ctsk, calcitonin receptor, and OC-associated receptor, through the synergistic activation of microphthalmia-associated transcription factor (Mitf), nuclear factor kappa B (NF-κB), c-Fos, and c-jun, thus affecting OC activity and regulating bone resorption (Yao et al., 2021). (Figure 4).

Treatment with Que reduces the expression of the NFATc1 gene and protein through the TRAF6/c-fos/NFATc1 signaling pathway and inhibits the differentiation of RAW264.7 cells to OCs (Liu et al., 2022). Que-3-O-β-D-glucoside inhibits OC differentiation by suppressing RANKL-induced NFATc1 expression, thereby preventing OC differentiation and bone loss (Shim et al., 2015).

#### 3.2.2 OPG/RANKL/RANK signaling pathway

The OPG/RANKL/RANK signaling pathway is a key signaling axis during bone remodeling (Yang et al., 2020; Velletri et al., 2021). In the presence of M-CSF, RANK binds to the C-terminus of RANKL and recruits tumor necrosis factor receptor-associated factors to induce transcription and expression of downstream OC-specific genes. It ultimately activates key transcription factors, such as NF-κB, activator protein 1, cyclic adenosine monophosphate, cyclic adenosine monophosphate response element binding protein, and NFATc1, and induces OC markers, such as TRAP, β3 integrin, and CtsK, which regulate OC differentiation and induce bone resorption (Belibasakis and Bostanci, 2012; Udagawa et al., 2021). OPG secreted by OB is a high-affinity decoy-like receptor for RANKL. OPG reduces the binding of RANKL to RANK and competitively inhibits the blocking of signals from OBs to OCs, thereby inhibiting OC generation and maturation and reducing OC activity (Ibrahim et al., 2016). (Figure 4).

Que upregulates the expression of OPG and downregulates the expression of RANKL in the femoral bone tissues of ovariectomized rats at both high and medium doses, inhibits bone resorption, prevents OP, and improves the biomechanical properties of the femur (Wang, 2009). Que can directly stimulate OPG expression in OVX rBMSCs while concurrently inhibiting RANKL expression. This dual action leads to the indirect increase of the OPG/RANKL ratio, reestablishing the balance of the RANKL/OPG system and restoring the healing capacity of damaged bone in the state of OP (Zhou et al., 2017). When OB-OC-endothelial cells were inoculated in three cultures on Que-containing hydroxyapatite, the trend of OPG/RANKL levels was similar to the reduction in histone K levels, suggesting that the presence of Que has an inhibitory effect on OC viability (Forte et al., 2016).

#### 3.2.3 ERK1/2/JNK signaling pathway

Among the OC precursors, two ERK forms (ERK1/2) and three JNK isoforms (JNK1/2/3) are mainly involved in OC precursor proliferation and OC apoptosis, respectively (Teitelbaum and Ross, 2003; Ikeda et al., 2008; Lee et al., 2016). ERKs have a typical protein kinase structure and control osteoclastogenesis by phosphorylating c-Fos, NFATc1, MITF, TFE3, hedgehog-Gli, Egr2, RSK2, and MMP-9 (Lee et al., 2018). JNK signaling regulates downstream c-Jun, CaMK, c-Fos, NFATc1, and semaphorin 3D to control OC metabolism (Noguchi et al., 2018). (Figure 4).

Que-3-O-β-D-glucuronide significantly attenuates the activation of JNK and ERK in LPS-stimulated RAW264.7 macrophages. It inhibits the secretion of plasmatic NO and PGE, as well as the expression of iNOS and COX-2, thus exerting an anti-inflammatory activity in a concentration-dependent manner (Park et al., 2016). Que exerts its anti-apoptotic effects through the JNK-c-Jun/AP-1 and ERK-c-Fos/AP-1 pathways (Ishikawa and Kitamura, 2000).

#### 3.2.4 Regulated inflammatory factors

Dynamic regulation of osteoclastogenic and anti-osteoclastogenic cytokines is essential for maintaining bone homeostasis (Amarasekara et al., 2018). During macrophage polarization, many inflammatory factors are involved in osteoclastogenesis through different pro-inflammatory and anti-inflammatory roles, further affecting the process of bone resorption and the development of OP (Pinti et al., 2016). Tumor necrosis factor-α (TNF-α) stimulates OC differentiation by upregulating RANK pro-inflammatory target genes through activating and inducing NF-κB nuclear translocation, disrupting the balance of the RANK-RANKL bio-axis, and increasing OC activity (Luo et al., 2018). Interleukin—1β (IL-1β) and IL-6 promote OC differentiation and maturation through a RANKL-independent mechanism, resulting in the occurrence of bone resorption (Duplomb et al., 2008; Ha and Choung, 2020). (Figure 4).

Que (2 or 5 μM) significantly reduces the accumulation of TNF-α and IL-6 in LPS-induced mouse RAW264.7 macrophages (Jung and Sung, 2004). Tsai et al. (2021) found that Que inhibits M1 polarization and significantly reduces the expression levels of M1 markers, such as IL-6, TNF-α, and IL-1β, in macrophages and microglia. Que significantly reduces the levels of TNF-α and IL-1β, inhibits OC activation, and attenuates bone destruction (Li et al., 2018).

## 4 Effects of Que in combination with other phytochemicals

Phytochemical treatments have been used to induce OB differentiation in in vitro models (Pan et al., 2005; Shakibaei et al., 2012; Zhou et al., 2015; Martiniakova et al., 2022; Shen et al., 2022; Zhou et al., 2023). The combination of Que with other bioactive ingredients has shown synergistic anti-osteoporotic effects (Rayalam et al., 2011). Giordani et al. (2023) found that a mixture of curcumin (1 M), polydatin (10 M), and Que (0.5 M) is a safe bioactive compound. This mixture has significant synergistic effects in promoting OB differentiation of MSCs and inhibiting inflammatory phenotypes associated with cellular senescence. It significantly reduces the expression of miR-21 and miR-146a, decreases the release of IL-8 and MCP-1, increases the expression of ALP mRNA, and efficiently reduces p38-MAPK and phosphorylated NF-κB. Lai et al. (2011) found that a combination of 2400 IU/kg of vitamin D, 400 mg/kg of resveratrol, 2,000 mg/kg of Que, and 1,040 mg/kg of genistein improves bone mineral density and trabecular structure and reduces postmenopausal bone loss in de-ovulated female rats. However, Ambati et al. (2018) found that the dietary intake of a combination of vitamin D, resveratrol, Que, and genistein, owing to their relatively low dose and synergistic properties, is not as effective as zoledronic acid in a postmenopausal rat model of OP. They reported that relatively low doses of phytochemicals may not have produced a sufficiently potent effect to prevent or reverse the dramatic loss of bone trabeculae induced by ovarian hormone deficiency. This lack of efficacy may be related to the prolonged duration of the study (16 weeks) and the dynamics of bone loss after estrogen withdrawal.

## 5 Pharmacokinetics of Que

Researchers have used high-performance liquid chromatography to determine the concentration of Que in various organ tissues. They found that Que is mainly distributed in the gastrointestinal tract by gastrointestinal ease of diffusion and absorption, followed by the blood, liver, kidney, heart, lungs, and spleen, and has a very low distribution in the brain and muscle tissues of rats (Meng and Wang, 2000; Wu et al., 2008). Que metabolites are excreted through the kidneys, feces, and the respiratory system (Moon et al., 2000; Russo et al., 2012). Que is rapidly metabolized in the blood and has a short half-life. Que metabolites are detected in plasma 30 min after ingestion, with the major metabolites being Que-30-sulfate, Que-3-glucuronide, and Que-3-sulfate. The highest concentrations are observed at 0.8 and 0.6 h, but they are excreted in large quantities within 24 h (Mullen et al., 2006; Moon et al., 2008). Que-30-glucuronide, Que-diuronate, isorhamnetin-sulfate glucuronate, isorhamnetin-methylquercetin, and isorhamnetin-diuronate are the major urinary metabolites, with the highest concentrations observed at 4 h (Moon et al., 2008).

Que exhibits low *in vitro* bioavailability (5.9%) and demonstrates solubility of 235.5 g/mL in water and 2.3 × 10^4^ g/mL in chloroform. It reaches a maximum concentration (Cmax) of 4.143 g/mL, an area under the curve of 12.015 g h/mL, and is encapsulated at a rate of 61%, with a drug loading capacity of 13 g/mg (Chen et al., 2019; Shao et al., 2019). Modern pharmaceutical scientists have improved the *in vitro* bioavailability of Que by applying delivery system technologies, such as particulate delivery systems, solid dispersions, encapsulation, phospholipid complexes, and hydrogels (Zhao et al., 2022). The amorphous solubility of Que-2-hydroxypropyl-β-cyclodextrin complex at small intestinal pH is at least 31 g/mL, with a Cmax value of 78.3 g/mL (Manta et al., 2020). The whey protein isolate-Que-lotus root branched chain amylose (LRA) hydrogel (whey protein isolate-Que-LRA) encapsulation rate was up to 92.4%, and Que was stable in the stomach and effectively released into the small intestine (Liu et al., 2020). The *in vitro* cumulative release of Que nanohybrids reached more than 65% at 30 min, which was significantly better than the cumulative release of Que APIs and physical mixtures at the same time (25.79% and 31.53%, respectively) (Liu et al., 2017). Shi et al. (2020) found a significant increase in the amount of Que passing through an artificial membrane after placing a Que/F68/HPMC 1/4/3 asd solution into the donor pool. Que shows a 12-fold increase in water solubility in the Que-phospholipid complex (from 3.44 μg/mL to 36.81 μg/mL) (Singh et al., 2012). Lee et al. (2020) loaded Que onto graphene planes by π-π stacking and weak hydrogen bonding. This loading method may result in a pH-responsive drug release mechanism in acidic environments, making it more suitable for targeted drug delivery in oncology therapy, with a maximum drug loading of 11 wt%. The oral administration of Que nanocapsules with triphenylphosphine cations as a matrix component with mitochondrial specificity results in high brain uptake and significant mitochondrial localization after cerebral ischemia/reperfusion. This effectively counteracts cerebral ischemia/reperfusion-induced cell death and neurodegenerative lesions in both young and aged rats (Ghosh et al., 2017).

## 6 Toxicology of Que

While some controversy exists regarding the safety of Que, the majority of views support the idea that Que is not toxic. Experimental studies of chronic toxicity have shown that after 2 years of administration of 0.1%, 1%, and 4% Que (equivalent to 40, 400, and 1900 mg/(kg·d)) in experimental rats, focal epithelial hyperplasia of the renal tubules was observed in male rats. The incidence of chronic kidney disease slightly increased with an increase in Que intake, and the incidence of renal adenomas was increased by 1% and 4% Que doses (Dunnick and Hailey, 1992). A reduction in body mass, an increased incidence of non-neoplastic polyp hyperplasia of the cecum, hyperplasia of the parathyroid glands in male rats, and the detection of calcium oxalate crystals in the urine were observed in rats following Que ingestion (Divi and Doerge, 1996; Andres et al., 2018). Que shows significant mutagenicity in mammalian cells and is potentially genotoxic (Huan et al., 2010). However, a 2-year rodent carcinogenicity bioassay conducted by the National Toxicology Program did not reveal any adverse effects relevant to the safety assessment of orally administered Que in humans (NTP, 1992; Harwood et al., 2007). Feng (2013) found that Que is free of acute toxicity, mutagenicity, and chronic or subchronic toxicity *in vivo* and *in vitro*. This finding was based on the acute toxicity test, Ames test, mouse bone marrow cell micronucleus test, mouse spermatozoa aberration test, the 30-day feeding test in rats, and the 56-day feeding test in laying hens. The State Food and Drug Administration (2017) published the World Health Organization’s International Agency for Research on Cancer list of carcinogens, classifying Que as a Group 3 carcinogen due to its suspected carcinogenicity to humans, but with insufficient human or animal data. The *in vitro* test lacks a well-established regulatory mechanism in the animal body. Therefore, no accurate animal test data have been established to support the conclusion that there is insufficient evidence to suggest that Que poses a safety risk. Therefore, based on the current animal safety evaluation tests within a reasonable dose range, the safety of Que is considered high.

## 7 Future perspective

Que, as a polyphenolic compound, does have some limitations in in vitro studies. One of the important limitations is its interfering effect on many assays, leading to false positive results (Baell and Holloway, 2010). Since Que has strong antioxidant, anti-inflammatory, anticancer and many other biological activities (Singh et al., 2021), it may affect certain biochemical reactions and assay results in in vitro experiments. Because of these interfering effects of Que, the reliability of data in in vitro studies is somewhat compromised. In order to reduce this interference, researchers should fully consider the effects of Que when conducting *in vitro* experiments and take appropriate measures to avoid or reduce the interference. For example, choosing appropriate experimental conditions, optimising experimental methods and reagents, setting up control groups, adding blocking agents, etc. In addition, clinical and *in vivo* studies can provide a more realistic evaluation of drug action effects and safety. Therefore, the results of clinical studies and *in vivo* studies should be combined when assessing the pharmacological effects and safety of Que to obtain a more comprehensive and accurate understanding. Also, when studying the interaction of Que with other drugs or compounds, attention needs to be paid to this interfering effect in order to avoid misleading conclusions.

The clinical applications of Que in the treatment of other diseases, such as type 2 diabetes (Mazloom et al., 2014) and obesity (Pfeuffer et al., 2013), have been explored and evaluated. Therefore, more high-quality large-sample clinical observations are warranted to discover and validate a more detailed mechanism of action for Que in the treatment of OP and to determine the optimal dosage, which will help promote its development as a functional food and drug.

However, several aspects must be considered during the development process. As the low solubility and bioavailability of Que limit its applications, researchers can improve its bioavailability through chemical modifications and composite carriers. In addition, intestinal flora was found to be involved in the development of OP (Hao et al., 2019; Zhang et al., 2021). How Que participates in bone metabolism by regulating the intestinal flora is not yet clear and will provide a new avenue for Que research.

## 8 Conclusion

This article reviews advances in the mechanisms, pharmacokinetics, and toxicology of Que for the treatment of OP. Que promotes OB-mediated bone formation mainly by regulating transcription factors, the Wnt/β-catenin signaling pathway, the BMP-2/SMADs/RUNX2 signaling pathway, anti-apoptosis-mediated pathways, and oxidative stress-mediated pathways, and by promoting bone matrix formation and mineralization. It also inhibits OC-mediated bone resorption by regulating transcription factors, inflammatory factors, the OPG/RANKL/RANK signaling pathway, and the ERK1/2/JNK signaling pathway. Thus, Que is a potential drug for the prevention and treatment of OP.
